# Beyond Therapy …

**DOI:** 10.1371/journal.pbio.0020181

**Published:** 2004-06-15

**Authors:** Robert Sinsheimer

## Abstract

In response to the Blackburn and Rowley essay on the President's Council on Bioethics, several thought-provoking opinions on ethical challenges in biomedical research are expressed by prominent stakeholders

It is indeed regrettable that a distinguished and thoughtful scientist such as Elizabeth Blackburn should have been dismissed from the President's Council on Bioethics. Scientific perspectives such as hers are surely needed on this committee.

Her dismissal was apparently the consequence of her disagreement with some of the text of the Council's report, “Beyond Therapy: Biotechnology and the Pursuit of Happiness” (2003). The thrust of this report is that some of the directions of current biological research will, if carried to fulfillment, result in major changes in the nature of human life—changes that the report regards with foreboding.

In their essay, Drs. Blackburn and Rowley (2004) try to bypass these concerns with the argument that we really are not able to accomplish any of these changes yet and, indeed, some may never be possible.

I would suggest that as scientists we should face these issues forthrightly. We should not seek refuge in presentday uncertainties. The authors of the report are not naïve nor ignorant. Yes, if these lines of research are successful, their outcome will change the nature of human life.

As an example, consider current research into the causes of aging. Clearly, we do not at present know how to achieve major increases in the human life span (although we are able to do so in lower life forms). But it is plausible that we will learn how to do so. And surely a, say, doubling of the human life span would change the nature of human life.

Likewise, if we learn to modify the human gene pool so as to produce exceptional individuals or to alter human capabilities, or if powerful drugs are developed that may commandeer the human psyche, the nature of human life will be altered.

But so be it. The nature of human life has changed repeatedly and profoundly in the past—with the invention of agriculture, with the invention of writing, with the development of machines and mechanical power, with the advent of modern science and medicine. The nature of human life is different in 2004 a.d. from what it was in 1000 a.d. or 46 b.c. or 5000 b.c. or 10,000 b.c., and it will change again in the future.

The concerns expressed in the report are earnest, and they should be confronted in earnest.

## References

[pbio-0020181-Blackburn1] Blackburn E, Rowley J (2004). Reason as our guide. PLoS Biol.

[pbio-0020181-PCB1] President's Council on Bioethics (2003). Beyond therapy: Biotechnology and the pursuit of happiness. http://bioethics.gov/reports/beyondtherapy/index.html.

